# Effect of C_60_ Fullerene on Recovery of Muscle Soleus in Rats after Atrophy Induced by Achillotenotomy

**DOI:** 10.3390/life12030332

**Published:** 2022-02-23

**Authors:** Dmytro Nozdrenko, Svitlana Prylutska, Kateryna Bogutska, Natalia Y. Nurishchenko, Olga Abramchuk, Olexandr Motuziuk, Yuriy Prylutskyy, Peter Scharff, Uwe Ritter

**Affiliations:** 1 Department of Biophysics and Medical Informatics, ESC “Institute of Biology and Medicine”, Taras Shevchenko National University of Kyiv, 01601 Kyiv, Ukraine; ddd@univ.kiev.ua (D.N.); bogutska_ki@knu.ua (K.B.); natalia.nuryschenko@knu.ua (N.Y.N.); 2 Department of Physiology, Plant Biochemistry and Bioenergetics, Faculty of Plant Protection, Biotechnology and Ecology, National University of Life and Environmental Science of Ukraine, 03041 Kyiv, Ukraine; psvit_1977@ukr.net; 3 Department of Human and Animal Physiology, Faculty of Biology and Forestry, Lesya Ukrainka Volyn National University, 43025 Lutsk, Ukraine; abramchuk.olga@vnu.edu.ua (O.A.); motuziuk.oleksandr@vnu.udu.ua (O.M.); 4 Institute of Chemistry and Biotechnology, Technical University of Ilmenau, 98693 Ilmenau, Germany; peter.scharff@tu-ilmenau.de

**Keywords:** muscle soleus of rat, achillotenotomy, atrophy, C_60_ fullerene, biomechanical and biochemical parameters of skeletal muscle contraction

## Abstract

Biomechanical and biochemical changes in the muscle soleus of rats during imitation of hind limbs unuse were studied in the model of the Achilles tendon rupture (Achillotenotomy). Oral administration of water-soluble C_60_ fullerene at a dose of 1 mg/kg was used as a therapeutic agent throughout the experiment. Changes in the force of contraction and the integrated power of the muscle, the time to reach the maximum force response, the mechanics of fatigue processes development, in particular, the transition from dentate to smooth tetanus, as well as the levels of pro- and antioxidant balance in the blood of rats on days 15, 30 and 45 after injury were described. The obtained results indicate a promising prospect for C_60_ fullerene use as a powerful antioxidant for reducing and correcting pathological conditions of the muscular system arising from skeletal muscle atrophy.

## 1. Introduction

Functional unloading of mammalian skeletal muscles caused by partial immobilization can cause their atrophy. In this case, the deepest atrophic changes are observed in the muscle soleus—the key postural muscle [1]. Possessing a pronounced plasticity, the skeletal muscle of mammals is able to rearrange its structural and metabolic profile, depending on the nature of contractile activity and changes in external conditions [2]. Regular strength training significantly increases the intensity of protein synthesis and, as a result, leads to hypertrophy of muscle fibers [3]. Conversely, functional unloading leads to suppression of protein synthesis and activation of proteolysis, which is reflected in a decrease in the diameter of muscle fibers (atrophy) and loss of their strength of contraction [4]. Muscle atrophy caused by prolonged inactivity is associated with both suppression of the intensity of protein synthesis and activation of intracellular proteolysis systems, which has been found in numerous animals and human model studies [5]. It has been shown that even short periods (5 days) of unuse of muscles can cause a significant loss of their mass and strength of contraction as well as accompaniment of physiological molecular rearrangements [6]. Both slow and fast fibers undergo atrophy; the largest fibers in an individual muscle usually show the greatest atrophic response. Atrophy of muscle fibers plateau after about 14 days of immobilization or gravitational inactivity. The increase in fatigue under these conditions reflects the loss of muscle and fiber mass. The glycolytic capacity of muscles and muscle fibers continues after immobilization for 30–40 days [7].

Since the initial discovery of MuRF1 and MAFbx as two muscle-specific E3 ubiquitin ligases, several additional mediators of muscle atrophy have been discovered, providing new insights into how muscle atrophy occurs at the molecular level [8]. Traditionally, two clinical models have been used to simulate unuse of the hind limbs in rats: Achilles tendon rupture (Achillotenotomy, AT) and suspension of the hind limbs. Significant atrophy of the gastrocnemius muscle occurs in both cases, starting at day 10 of the study. Degradation of the basement membrane of muscle fibers leads to impaired muscle contractility. A significant decrease in the force of contractions of the gastrocnemius muscle, isometric tetanic force and the rate of contraction after tendon rupture has been demonstrated [9]. Achilles tendon rupture and subsequent muscle atrophy leads to functional impairments, which can also be caused by morphological changes in the muscle–tendon block. The functional characteristics of the injured limb will be impaired regardless of the time that has elapsed after the operation, and these impairments occur together with changes in the morphology of the muscle–tendon. Disorders can persist for many years in the postoperative period, although they can be more pronounced with high-speed activity [10] and disrupt nonlinear processes in muscle biomechanics [11].

Selection of AT as a model for imitation of unuse of rats’ hind limbs is based on its weightier social significance. Acute rupture of the Achilles tendon is a common injury that can lead to disability. Over the past decade, in the treatment of acute rupture of the Achilles tendon and the resulting atrophy of muscle groups, there has been a transition from surgical treatment to non-surgical treatment. However, the optimal protocol for non-surgical treatment is under development [12]. The problem of increasing the length of the Achilles tendon after its rupture remains unresolved, which is associated with a decrease in the volume of the calf muscles and a persistent deficit in plantar flexion strength after surgical recovery. The deficit in muscle strength and volume is partially compensated by hypertrophy: a deficit in muscle soleus volume from 11% to 13% and a deficit in plantar flexion strength from 12% to 18% persist even after long-term follow-up [13]. Muscle atrophy, joint stiffness, osteoarthritis, infection, necrosis and ulceration of the articular cartilage are known complications caused by prolonged immobilization of surgically repaired Achilles tendon ruptures [14].

The main methods of treating such injuries, based on the surgical repair of tendon ruptures, have a number of significant drawbacks [15]. Serious degradation of the muscular apparatus always occurs during therapeutic procedures and the rehabilitation period of recovery [16]. Thus, the development of a rehabilitation protocol is an essential aspect of restoration of the pre-injury activity levels. Despite several available trials, which compare different treatment regimens, there is still no consensus on the optimal protocol [17].

Recently, the use of antioxidant therapy in the early stages of the development of muscle atrophy demonstrates the promise of this approach. The authors of [18] showed that the use of an antioxidant, curcumin, leads to a decrease in oxidative stress and the activity of proteolytic pathways and, as a consequence, decreases the degradation of muscle protein during the development of muscle atrophy. It has been shown that licorice flavonoid oil that contains glabridin, and exhibits strong antioxidant properties, increases muscle mass in mice with muscle atrophy. Oral administration of glabridin prevented induced protein degradation in the tibialis anterior muscle of mice. This indicates that the antioxidant glabridin is an effective food ingredient for preventing skeletal muscle atrophy [19]. At the same time, no advantages of oral administration of the studied additives in collagen synthesis or improvement of the biomechanical properties of atrophied muscles were found after 3 weeks of use while studying the effect of vitamin C on the healing of the Achilles tendon in rats. Therefore, the search for an optimal antioxidant for the treatment of muscular atrophy is still ongoing [20].

It is known that C_60_ fullerene is capable of inactivating methyl, superoxide anion and hydroxyl radicals in in vivo and in vitro systems [21,22]. In our previous studies, it was shown that administration of water-soluble C_60_ fullerenes after initiation of ischemic damage leads to significant positive therapeutic effects [23]. A positive trend has been shown when using them for muscle injury [24], muscle dysfunction associated with pesticide poisoning [25], as well as the development of fatigue processes [26]. All these data stimulated us to test water-soluble C_60_ fullerenes as potential therapeutic agents that reduce pathological effects in the muscular system of rats during the development of AT-associated dystrophy.

## 2. Materials and Methods

The experiments were performed on male Wistar rats aged 2 months weighing 200 ± 6 g. The study protocol was approved by the bioethics committee of the ESC Institute of Biology and Medicine, Taras Shevchenko National University of Kyiv in accordance with the rules of the European Convention for the Protection of Vertebrate Animals Used for Experimental and Other Scientific Purposes and the norms of biomedical ethics in accordance with the Law of Ukraine №3446–IV 21.02.2006, Kyiv, on the Protection of Animals from Cruelty during medical and biological research.

Before the start of the study, rats underwent Achilles tendonomy—a cut of the Achilles tendon. The following groups of animals were studied: intact group of animals (n = 7), groups of animals on days 15, 30 and 45 after AT without administration of water-soluble C_60_ fullerenes (n = 7 in each group) and with administration of water-soluble C_60_ fullerenes (n = 7 in each group). In preparation for the experiment, anesthesia of animals was performed by intraperitoneal administration of nembutal (40 mg/kg). The standard preparation included the cannulation (a. carotis communis sinistra) for pressure measurement and laminectomy at the lumbar spinal cord level. muscle soleus of rat was released from the surrounding tissues. Its tendon was cut across the distal part, which was connected to the force sensors. For modulated stimulation of efferents, the ventral roots were cut at the points of their exit from the spinal cord. Research of muscle contraction dynamics was performed under the conditions of muscle activation using the method of modulated efferent stimulation [27]. Filaments of the cut ventral roots were fixed on the stimulating electrodes and cyclic distribution of the stimulus sequence was performed. Stimulation of efferents was performed by electrical pulses lasting 2 ms, generated by the impulse generator. The control of the external load on the muscle was performed using a system of mechanical stimulators. The change in force was measured using strain gauges.

In the process of analyzing the obtained results, the integrated muscle power (calculated area under the force curve) was used as parameter, which is an indicator of the general performance of the muscle with the applied stimulation pools [28]. The development of muscle contractile activity was assessed by the method of calculating time intervals when 50% of the levels of strength responses were reached during stimulation.

An aqueous colloidal solution of C_60_ fullerenes was obtained using the original ultrasonic technology [29,30]. At a maximum concentration of 0.15 mg/mL, it remains stable for 18 months at a storage temperature of +4 °C.

The data of the authors [31] show that the time before the onset of atrophy caused by muscle unloading is the most optimal for therapeutic intervention in preventing skeletal muscle atrophy, which is associated with the redox balance. Based on this, the protocol of our research assumed the initiation of the administration of water-soluble C_60_ fullerenes immediately after the initiation of the injury.

Water-soluble C_60_ fullerene was administered orally at a dose of 1 mg/kg each day of the experiment. An appropriate amount of the solution was poured into a rat drinker, each of which was kept in a separate cage. Further feeding and watering of the animal was carried out only after the emptying of the drinker.

It is important to note that the selected dose of water-soluble C_60_ fullerene in our experiments is significantly lower than the LD_50_ value, which was 600 mg/kg body weight when administered orally to rats [32] and 721 mg/kg when administered intraperitoneally to mice [33].

The level of enzymes content in the blood of experimental animals, namely, the thiobarbituric acid reactive substances (TBARS), hydrogen peroxide (H_2_O_2_), reduced glutathione (GSH) and catalase activity (CAT)), as markers of muscle injury, was determined using clinical diagnostic equipment—a haemoanalyzer [25].

Single muscle fibers were isolated surgically using microsurgical instruments under a binocular microscope. In each experiment, we removed the muscle soleus, which was dissected in its thickest part (2/5 of its length from the proximal end). A Nikon inverted microscope (×600) and a Panasonic video camera system were used to determine the diameter of a single soleus fiber. Fiber diameter was determined using a calibration eyepiece as the average of three measurements and, accordingly, the cross sectional area (CSA) was calculated.

Each of the experimental curves shown in the figures is the result of averaging 10 similar tests. The same averaging proportions were used in each of the groups of animals studied. Statistical processing of measurement results was performed by methods of variation statistics using software Original 9.4.

Data are expressed as the means ± SEM for each group. The differences among experimental groups were detected by one-way ANOVA followed by Bonferroni’s multiple comparison test. Values of *p* < 0.05 were considered significant.

## 3. Results and Discussion

### 3.1. Dynamics of Muscle Soleus Contraction Force in Rats

**Figure 1:**
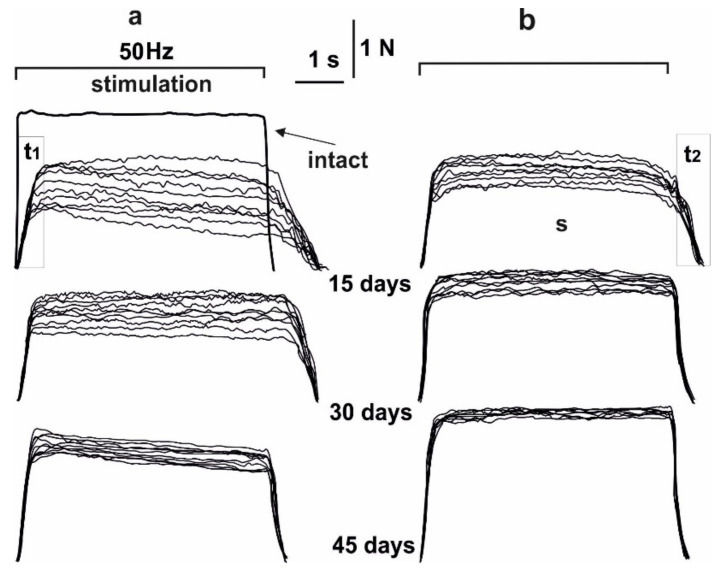
The force of contraction of the muscle soleus after AT in rats, caused by 10 consecutive 6 s non-relaxation pools of stimulation: without C_60_ fullerenes administration (**a**); with C_60_ fullerenes administration at a dose of 1 mg/kg (**b**). Native muscle–intact, t_1_–time of the maximum strength response development, t_2_–recovery time of strength parameters to their initial values, S–integrated power of muscle contraction, calculated as the total area under the corresponding strength curve.

**Figure 2:**
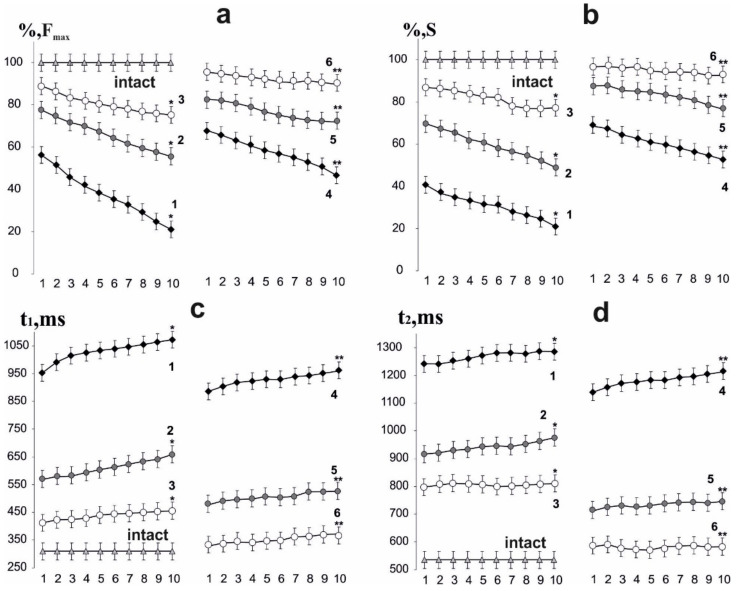
Biomechanical parameters of muscle soleus after AT in rats at 10 consecutive 6 s non-relaxation contractions: change in the maximum force response as a percentage of the values in the intact group (**a**); integrated muscle power as a percentage of values in the intact group (**b**); time of development of the maximum force response (**c**); recovery time of force parameters to their original values (**d**). Native muscle–intact; 1,2,3–the values of the corresponding parameters on 15th, 30th and 45th days after AT, respectively, without administration of C60 fullerenes (*p < 0.05 compare to the intact group at all 1, 2,…10 consecutive contractions); 4,5,6–the values of the corresponding parameters on 15th, 30th and 45th days after AT, respectively, after using C60 fullerenes at a dose of 1 mg/kg (**p < 0.05 compared to the group without the use of C60 fullerene at all 1, 2,…10 consecutive contractions).

On day 15 after AT initiation, the maximal muscle soleus contraction force of rats induced by 6 s with nonrelaxation stimulation pools decreased to 58 ± 2% in the first contraction and to 23 ± 5% in the tenth, relative to the intact group. Thus, there was a sharp decrease in muscle strength activity already at the first contractions with a progressive decrease in the studied biomechanical parameters (Figure 1 and Figure 2). On days 30 and 45 after AT, the maximal force response decreased to 79 ± 5% in the first reduction and 59 ± 4% in the tenth and to 88 ± 7% in the first reduction and 78 ± 7% in the tenth, respectively. After using C_60_ fullerene therapy, these values were 65 ± 6% in the first reduction and 45 ± 6% in the tenth, 84 ± 7% in the first reduction and 77 ± 3% in the tenth, 95 ± 9% in the first reduction and 91 ± 5% at the tenth on days 15, 30 and 45 after AT, respectively. The therapeutic effect averaged 45–55%.

The decrease in the integrated power of muscle contraction on the 15th day after initiation of AT was 41 ± 2% after the first contraction and 22 ± 4% after tenth, respectively, relative to the intact group. On days 30 and 45, these indicators were 70 ± 3% and 53 ± 4%, 84 ± 7% and 79 ± 7% after first and tenth contractions, respectively. These indicators were 73 ± 3% and 59 ± 7%, 85 ± 6% and 78 ± 7%, 94 ± 3% and 92 ± 6% after the first and tenth contractions, respectively, using C_60_ fullerene therapy on days 15, 30 and 45 after AT. The therapeutic effect averaged 35–40%.

### 3.2. Estimation of the Time to Reach the Maximum Force Response and Recovery of Muscle Soleus Force Parameters in Rats

The time to reach the maximum force response is one of the most important biomechanical parameters, since its change significantly affects the quality of targeted movements and the adequate implementation of motoneuronal pools. On the 15^th^ day after AT activation, an increase in this indicator was recorded from 961 ± 5 ms after the first contraction to 1070 ± 7 ms after the tenth, in comparison with the intact group (275 ± 9 ms). On days 30 and 45 after AT activation, these indicators were 570 ± 11 and 660 ± 14 ms, 400 ± 7 and 445 ± 7 ms after the first and tenth contractions, respectively. After using C_60_ fullerene therapy, a correction of these parameters was recorded: 872 ± 12 and 954 ± 8 ms, 460 ± 13 and 524 ± 12 ms, 336 ± 14 and 378 ± 12 ms after the first and tenth contractions on days 15, 30 and 45, respectively. The therapeutic effect averaged 50–60% on the 15th day after AT and 20–25% after 45 days. This can be explained by the fact that pathological factors affecting the time to reach the maximum force response are on the first days after AT and decrease their pathological effect with an increase in the time after the described injury. The progressive decrease in the force response lasts at least 15 days, after which the recovery process takes place [34].

The recovery time of force parameters to their initial values is directly affected by an increase in muscle stiffness and a change in the elastic properties of tendon components. On the 15th day after AT activation, its increase was recorded as 1240 ± 58 ms after the first contraction and 1290 ± 15 ms after the tenth in comparison with intact group (521 ± 16 ms). On days 30 and 45, these indicators were 900 ± 16 ms and 993 ± 21 ms, 790 ± 17 and 800 ± 18 ms after the first and tenth contractions, respectively. Its slight growth with an increase in the number of 6 s non-relaxation contractions against its significant decrease with increasing of time after AT should be noted. With the use of C_60_ fullerene therapy, a significant decrease in the recovery time of force parameters to the initial values was recorded: 1123 ± 19 and 1211 ± 15 ms, 722 ± 18 and 749 ± 13 ms, 590 ± 24 and 593 ± 19 ms after the first and tenth reduction on the 15, 30 and 45 days after AT, respectively. Thus, the obtained data indicate a positive dynamic of the therapeutic use of water-soluble C_60_ fullerenes in a daily dose of 1 mg/kg, which leads to a decrease in the level of muscle damage severity by an average of 25–35%.

### 3.3. Analysis of Fatigue Processes in the Muscle Soleus of Rats after AT Using 1 Hz Stimulation

**Figure 3:**
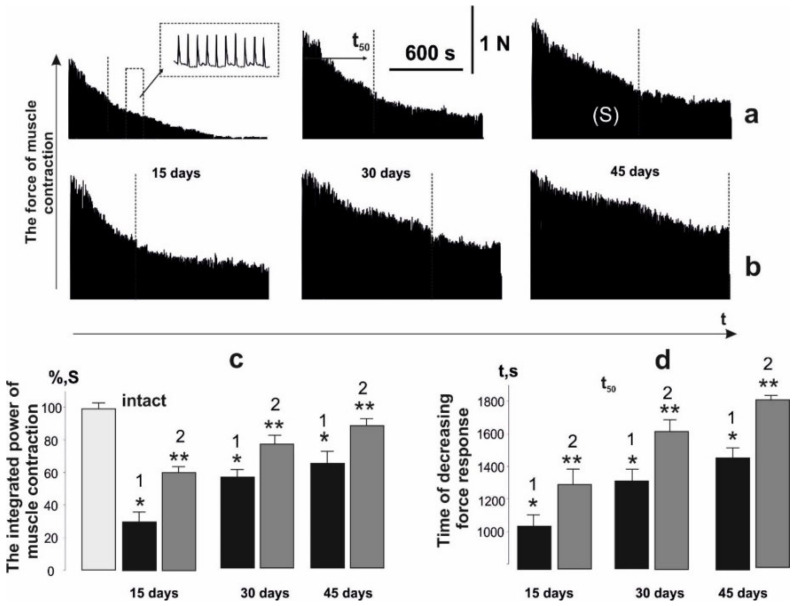
Biomechanical parameters of muscle soleus after AT in rat at 1 Hz stimulation for 1800 s: without C_60_ fullerenes administration (**a**); with the use of C_60_ fullerenes at a dose of 1 mg/kg (**b**); integrated muscle power (S), presented as a percentage of values in the intact group (**c**); time reduction of the force response by 50% from the initial values (t_50_) (**d**). Native muscle–intact; 1,2–the corresponding values of the parameters without and with C_60_ fullerenes use, respectively. *p < 0.05 compare to the intact group; **p < 0.05 compare to the group without the use of C_60_ fullerene.

Previously, an increase in the amount of intramuscular connective tissue due to trauma was revealed, which, apparently, occurs simultaneously with muscle atrophy and loss of muscle capillarity [35]. These factors are key to the onset of increased muscle fatigue in the active muscle. Therefore, the next stage of our research was to analyze the occurrence of the fatigue processes in the muscle soleus after AT upon application of stimulation. Registration of the contraction force with the use of 1 Hz stimulation for 1800 s showed a decrease in the integrated muscle power (Figure 3): it was 28 ± 2%, 59 ± 6% and 64 ± 4% relative to the intact group on days 15, 30 and 45 of the experiment, respectively. The use of water-soluble C_60_ fullerenes improved this indicator to 61 ± 2%, 78 ± 4% and 88 ± 7% on days 15, 30 and 45 of the experiment, respectively. The therapeutic effect was more than 50%, which may be due to the antioxidant properties of C_60_ fullerenes to correct fatigue processes in the active muscle [36].

The time for force response to decrease by 50% of the initial values (t_50_) without C_60_ fullerene therapy was 1020 ± 42, 1310 ± 65 and 1490 ± 85 ms on days 15, 30 and 45 of the experiment, respectively. After using of water-soluble C_60_ fullerenes this indicator was 1325 ± 72, 1680 ± 77, and 1780 ± 59 ms, respectively, which shows its 50% therapeutic effect at the stages of maintaining the maximum force responses during the development of fatigue processes.

### 3.4. Analysis of the Occurrence of Smooth Tetanic Contraction of Muscle Soleus in Rats

The most important quantitative indicator of skeletal muscles work in the process of functioning is the rate of smooth tetanic contraction occurrence. Even minimal physiological or biochemical destructive changes in the structure of myocytes and motoneuronal pools, changes in muscle stiffness and electrical properties of membranes or the duration of hyperpolarization significantly change the time of smooth tetanic contractions occurrence [37]. Moreover, during muscle activity, its individual motor units generate unfused tetanic contractions, which are characterized by variable strength and varying degrees of fusion. The synchronization of this process depends on many factors and is a vulnerable element in the development of pathological processes in the muscle [38]. Therefore, the next step was to study biomechanical markers of the appearance of smooth tetanic contractions.

**Figure 4:**
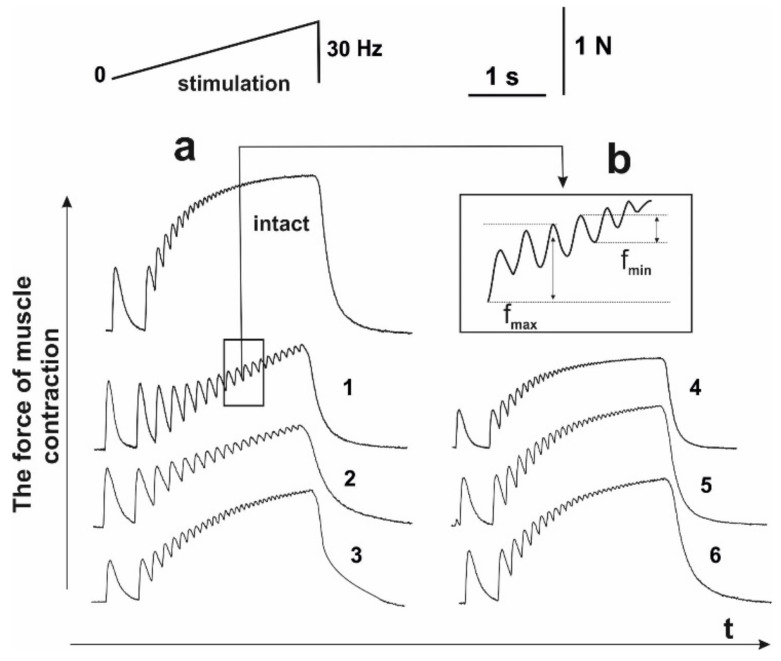
Mechanograms of the transition of muscle soleus after AT in rats from dentate to smooth tetanus with the use of increasing stimulation with a maximum frequency of 30 Hz for 6 s: without C_60_ fullerenes administration (**a**); with C_60_ fullerenes administration in a daily dose of 1 mg/kg (**b**). Native muscle–intact; f_max_ is the maximum force of a single contraction, f_min_ is the minimum value of the force response in one tooth of the dentate tetanus; 1,2,3–the values of the corresponding parameters on 15, 30 and 45 days after AT, respectively, without C_60_ fullerenes administration; 4,5,6–the values of the corresponding parameters on 15, 30 and 45 days after AT, respectively, with the use of C_60_ fullerenes at a dose of 1 mg/kg.

**Figure 5:**
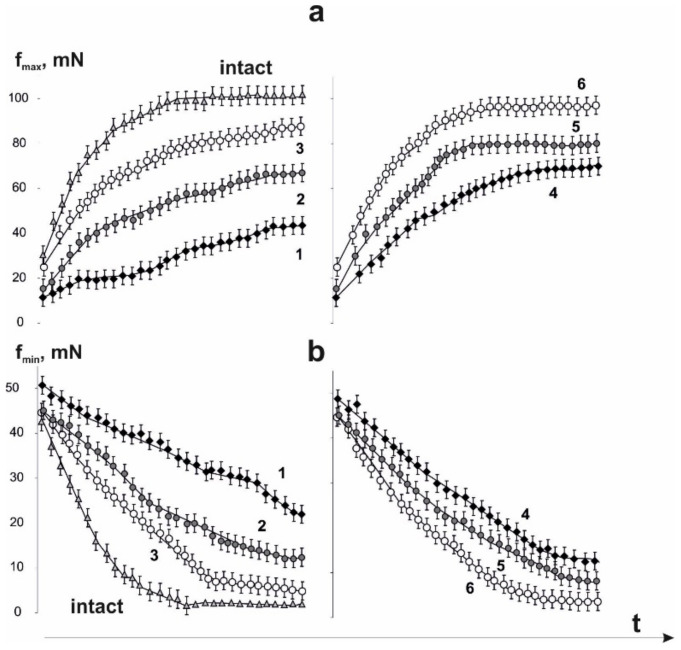
Changes in f_max_ (**a**) and f_min_ (**b**) parameters of muscle soleus after AT for each of the single contractions during the transition of the force response to smooth tetanus using an increasing stimulation signal with a maximum frequency of 30 Hz for 6 s: 1,2,3–parameter values on days 15, 20 and 45 after AT, respectively, without C_60_ fullerenes administration; 4,5,6–the values of the parameters on days 15, 20 and 45 after AT, respectively, with the use of C_60_ fullerenes at a dose of 1 mg/kg.

Using of stimulation pools with increasing frequency (Figure 4), the smooth tetanic contractions (maximum force response) appeared after 3450 ± 12 ms and reached 97 ± 8 mN (Figure 5). Muscle soleus after AT did not reach the stage of smooth tetanic contraction throughout the experiment. The maximum force of a single contraction (f_max_) was 43 ± 2, 67 ± 4, and 87 ± 2 mN on days 15, 30 and 45 of the experiment, respectively. The use of water-soluble C_60_ fullerenes increased these indicators to 72 ± 3, 79 ± 5, and 94 ± 2 mN on days 15, 30 and 45 of the experiment, respectively. The minimum value of the force response in one tooth of dentate tetanus (f_min_) slightly decreased to 22 ± 3, 17 ± 2 and 5 ± 1 mN on days 15, 30 and 45 of the experiment, respectively. It should be noted that a decrease in this parameter to zero leads to the appearance of smooth tetanus. The use of C_60_ fullerene changed the biomechanical parameters of the transition of muscle soleus from dentate to smooth tetanus, which appeared 4350 ± 32 and 3650 ± 32 ms on days 30 and 45 after AT, respectively. All the described biomechanical parameters after the application of C_60_ fullerene showed positive therapeutic dynamics at the level of 23–29%.

### 3.5. Changes in the Body Weights of Animals and the Muscle Soleus, the Value of the Maximum Strength of a Single Tetanic Contraction of an Isolated Muscle, Normalized to the Value of CSA, after AT

The weight of rats of all groups slightly increased during the experiment; this change was taken into consideration for further calculations (Table 1). The mass of muscle soleus normalized to the body weight significantly decreased to 0.27 ± 0.032 g on the 15th day after AT and increased to 0.34 ± 0.018 g on the 45th day (in comparison with intact group, this value was 0.49 ± 0.011 g). In the groups that received C_60_ fullerene, these indicators were 0.32 ± 0.015, 0.35 ± 0.023 and 0.39 ± 0.054 g on days 15, 20 and 45 after AT, respectively, which is on average 35–37% higher than in the previous group.

The maximum strength of a single tetanic contraction (P_0_) (this value in the intact group was 882.4 ± 14.3 mN) decreased to 432.5 ± 16.1, 676.5 ± 11.6 and 693.3 ± 14.1 mN on days 15, 30 and 45 after AT, respectively. The use of C_60_ fullerene improved this indicator to 602.5 ± 12.2, 711.5 ± 22.5 and 782.5 ± 16.3 mN on days 15, 30 and 45 after AT, respectively, which showed an increase in the P_0_ value by more than 30%. The most significant results were shown by changes in the maximum strength of a single tetanic contraction (P_0_), normalized to the value of CSA. The decrease in P_0/_CSA value to 14.4 ± 2.5, 17.6 ± 7.3 and 18.6 ± 4.4 N/cm^2^ on days 15, 30 and 45 after AT was 61.5, 75.2 and 78%, respectively, in comparison with the value in the intact group (23.4 ± 1.2 N/cm^2^). With the use of C_60_ fullerene, these indicators were 18.1 ± 1.2, 19.2 ± 1.1 and 20.3 ± 1.2 N/cm^2^ on 15, 30 and 45 days after AT, respectively, which is more than 40% higher than in the previous group. According to the obtained data, it can be concluded that C_60_ fullerenes administration in a daily dose of 1 mg/kg reduces the level of destruction of muscle tissue by 30–35%.

### 3.6. Analysis of Blood Biochemical Parameters in Rats as Markers of Muscle Injury

**Figure 6:**
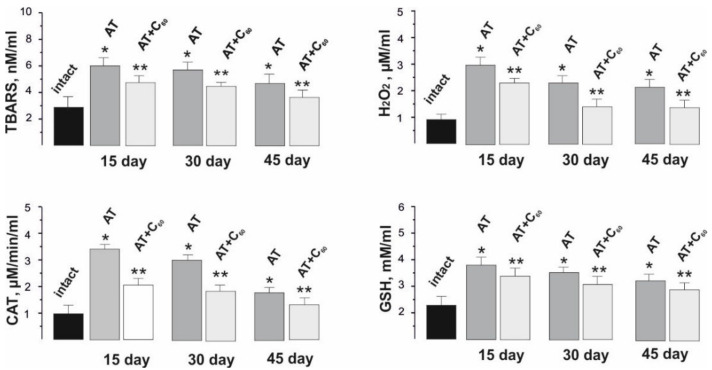
Indicators of pro- and antioxidant balance (TBARS, H_2_O_2_, CAT and GSH) in the blood of rats after 1 Hz stimulation of muscle soleus for 1800 s on 15, 20 and 45 days after AT. * *p* < 0.05 compare to the intact group; ** *p* < 0.05 compare to the group without C_60_ fullerenes administration.

Unused muscles atrophy is part of numerous pathologies in which the loss of muscle mass ultimately leads to the depletion of the organism (cachexia). Whether it is caused by muscle failure or disease, muscle loss results in weakness and metabolic co-morbidity. Reactive oxygen species (ROS) are important regulators of cellular signaling pathways that can accelerate proteolysis and suppress protein synthesis [39]. The authors of [40] showed that increased production of ROS in skeletal muscles significantly contributes to their atrophy caused by inactivity. Inflammatory cascade processes that occur immediately after AT are a source of ROS and contribute to the intensification of lipid peroxidation (LPO) processes. As a result of biochemical tests, we determined the levels of LPO secondary products and antioxidants in the blood of rats after AT. The obtained data clearly demonstrate an increased level of markers of peroxidation and oxidative stress (CAT, H_2_O_2_, TBARS and GSH) after AT and their decrease after C_60_ fullerene therapy (Figure 6).

Thus, the CAT level increased from 0.9 ± 0.1 µM/min/mL (in the intact group) to 3.5 ± 0.3, 3.1 ± 0.4 and 1.8 ± 0.6 µM/min/mL on days 15, 30 and 45 after AT, respectively, and decreased to 2.0 ± 0.4, 1.8 ± 0.1 and 1.3 ± 0.5 μM/min/mL on days 15, 30 and 45 after AT with the use of C_60_ fullerene therapy, respectively. The level of H_2_O_2_ was 2.8 ± 0.3, 2.5 ± 0.3, and 2.3 ± 0.6 μM/mL on days 15, 30 and 45 after AT, respectively (in the intact group, this value was 0.8 ± 0.2 µM/mL), and decreased to 2.1 ± 0.7, 1.5 ± 0.2 and 1.4 ± 0.6 µM/mL on days 15, 30 and 45 with the use of C_60_ fullerene therapy, respectively. The TBARS level was 6.1 ± 0.1, 5.5 ± 0.8 and 4.3 ± 0.3 nM/mL on days 15, 30 and 45 after AT, respectively (in the intact group, this value was 2.9 ± 0.2 nM/mL), and 4.3 ± 0.4, 4.1 ± 0.8 and 3.8 ± 0.6 nM/mL on days 15, 30 and 45 after AT with the use of C_60_ fullerene therapy, respectively. The GSH concentration was 3.8 ± 0.4, 3.4 ± 0.2 and 3.2 ± 0.3 mM/mL on days 15, 30, and 45 after AT, respectively (in the intact group, this value was 2,0 ± 0.3 mM/mL), and 3.3 ± 0.2, 3.1 ± 0.7 and 2.9 ± 0.3 mM/mL on days 15, 30 and 45 after AT with the use of C_60_ fullerene therapy, respectively.

Thus, there is a positive change in the described biochemical parameters by approximately 27–30% after therapeutic administration of C_60_ fullerene. This indicates the presence of compensatory activation by C_60_ fullerene of the endogenous antioxidant system in the process of dystrophic changes in the muscle soleus caused by AT. In our opinion, C_60_ fullerene can affect the activity of endogenous antioxidants, suppressing the occurrence of destruction in the muscle and, thus, reducing its degradation. The therapeutic effect of water-soluble C_60_ fullerenes on the restoration of tendon structures is also possible, this was confirmed by the previously obtained data about their protective effect in inflammatory and pathological processes in the body [41,42,43].

Despite the fact that atrophy that occurs after traumatic joint injury has morpho-functional differences against muscle atrophy that develops as a result of their unuse, the main mechanisms leading to changes in muscle mass in this pathology do not differ significantly [44]. Therefore, it can be assumed that there is not a significant difference in the treatment of atrophic pathologies caused by these factors.

## 4. Conclusions

Based on the obtained data, we can conclude that the positive therapeutic changes in the studied biomechanical and biochemical markers confirm the possibility of using water-soluble C_60_ fullerene (oral administration at a dose of 1 mg/kg each day of the experiment) as a promising nanoagent that can reduce and correct pathological states of the muscular system arising from skeletal muscle atrophy due to unuse.

## Figures and Tables

**Table 1:** Changes in the weight of the animals’ bodies and muscle soleus, the values of the maximum strength of a single tetanic contraction of an isolated muscle (P_0_) and P_0/_CSA on days 15, 30 and 45 after AT.

Group	Rat Weight, g	Soleus Weight, mg	Soleus Weight/Rat weight	P_0_, mN	P_0_/CSA, N/cm^2^
intact	205 ± 8	102.4 ± 1.5	0.49 ± 0.011	882.4 ± 14.3	23.4 ± 1.2
15 days	231 ± 6 *	63.4 ± 1.8 *	0.27 ± 0.032 *	432.5 ± 16.1 *	14.4 ± 2.5 *
30 days	243 ± 4 *	73.4 ± 1.2 *	0.30 ± 0.015 *	676.5 ± 11.6 *	17.6 ± 7.3 *
45 days	250 ± 6 *	86.4 ± 1.5 *	0.34 ± 0.018 *	693.3 ± 14.1 *	18.6 ± 4.4 *
15 days + C_60_ fullerene	244 ± 5 **	79.4 ± 1.2 **	0.32 ± 0.015 **	602.5 ± 12.2 **	18.1 ± 1.2 **
30 days + C_60_ fullerene	254 ± 2 **	89.4 ± 1.3 **	0.35 ± 0.023 **	711.5 ± 22.5 **	19.2 ± 1.1 **
45 days + C_60_ fullerene	269 ± 7 **	105.4 ± 1.9 **	0.39 ± 0.054 **	782.5 ± 16.3 **	20.3 ± 1.2 **

* *p* < 0.05 compare to intact group; ** *p* < 0.05 compare to the group without C_60_ fullerene administration.

## Data Availability

Data can be obtained upon request to the authors.
